# Transparency in medical error disclosure: the need for formal teaching in undergraduate medical education curriculum

**DOI:** 10.3402/meo.v19.23542

**Published:** 2014-01-31

**Authors:** Lucman A. Anwer, Ahmed Abu-Zaid

**Affiliations:** College of Medicine, Alfaisal University, Riyadh, Saudi Arabia

Timely and explicit medical error disclosure is essential to maintain a strong bond of trust between physicians and their patients. Several surveys revealed that patients desire to be informed promptly of all medical errors (including the unintended minor ones) (1, 2), and furthermore, prefer to be debriefed in greater details than what most physicians think is needed (3). However, there is a wide gap between patients’ demands of transparency in disclosing all medical errors and attempts of healthcare providers to do so. A recent study revealed that in hypothetical situations, 90% of the healthcare providers (medical students, residents, and physicians) stated that they would disclose medical errors; however, in real-life circumstances, only 41% actually reported doing so (4). A genuine question arises: What is the reason behind this ‘demand and supply’ mismatch?

Worldwide, medical errors are a disappointing, yet an unavoidable reality of the healthcare system, and there is unquestionably a high probability that medical students and residents will encounter such incidents during their training. The main dilemma, however, does not lie in the errors themselves, but rather in their full transparent disclosure. Despite the desires of medical students and residents to be open to patients and disclose medical errors, many are not sufficiently well prepared to deal with such situations (5). This is primarily because ‘formal’ instruction of transparency in medical error disclosure, a fundamental professional skill, is largely negligible and not adequately instructed in the vast majority of undergraduate medical education curricula (6–8). Hence, the poorly developed competency of professional medical error disclosure remains a ‘weakness’ as undergraduate medical students advance in their medical education and manifests in its full-blown picture during residency training when they bear far greater patient responsibilities.

As a direct consequence of this near-absent teaching, many physicians-in-training (medical students, interns, and residents) are confronted with distressing challenges when they face disclosure of medical errors to patients or healthcare institutes (5). This unconsciously compels them to follow their pure gut feeling (as opposed to knowledge-based inclinations) in handling such situations by attempting to ‘conceal’ those medical errors (because of fear of lawsuit litigations, etc.) (5), which often results in further harm to patients and healthcare institutes.

As ‘practice makes perfect’, we believe that the incorporation of formal teaching of transparent medical error disclosure in medical curricula is greatly needed. Medical schools play central roles in cultivating the significance and developing the communication skills needed for proficient and effective medical error disclosure. Moreover, they play key roles in resolving all barriers that may hinder transparency and full disclosure of medical errors. Such an approach is expected to educate a safe physician workforce where intrinsic drives and capabilities to remain transparent at all times – regardless of consequences – will serve as the basis for enhancing patient–doctor relationships, limiting further harm and improving overall healthcare safety (9).
